# Sodium compensation: a critical technology for transforming batteries from sodium-starved to sodium-rich systems

**DOI:** 10.1039/d4sc03995e

**Published:** 2024-08-09

**Authors:** Bin Zhu, Wei Zhang, Zhenjing Jiang, Jie Chen, Zheng Li, Jingqiang Zheng, Naifeng Wen, Ruwei Chen, Hang Yang, Wei Zong, Yuhang Dai, Chumei Ye, Qi Zhang, Tianyun Qiu, Yanqing Lai, Jie Li, Zhian Zhang

**Affiliations:** a School of Metallurgy and Environment, Engineering Research Center of the Ministry of Education for Advanced Battery Materials, Hunan Provincial Key Laboratory of Nonferrous Value-Added, Metallurgy, Central South University Changsha 410083 P. R. China zhangzhian@csu.edu.cn; b Department of Chemistry, University College London London WC1H 0AJ UK wei.zhang.21@ucl.ac.uk; c Department of Engineering Science, University of Oxford Oxford OX1 3PJ UK; d Department of Materials Science and Metallurgy, University of Cambridge Cambridge CB3 0FS UK; e School of Engineering, University of Warwick Coventry CV4 7AL UK; f School of Photovoltaic and Renewable Energy Engineering, University of New South Wales Sydney NSW 2052 Australia; g Department of Chemical System Engineering, School of Engineering, The University of Tokyo Hongo 7-3-1 Bunkyo-ku Tokyo 113-8656 Japan

## Abstract

Sodium-ion batteries (SIBs) have attracted wide attention from academia and industry due to the low cost and abundant sodium resources. Despite the rapid industrialization development of SIBs, it still faces problems such as a low initial coulombic efficiency (ICE) leading to a significant decrease in battery energy density (*e.g*., 20%). Sodium compensation technology (SCT) has emerged as a promising strategy to effectively increase the ICE to 100% and drastically boost battery cycling performance. In this review, we emphasize the importance of SCT in high-performance SIBs and introduce its working principle. The up-to-date advances in different SCTs are underlined in this review. In addition, we elaborate the current merits and demerits of different SCTs. This review also provides insights into possible future research directions in SCT for high-energy SIBs.

## Abstract 2

This review highlights the significance, fundamental principle, and recent advances of sodium compensation technologies, and provides insights into future improvement.

## Graphical abstract

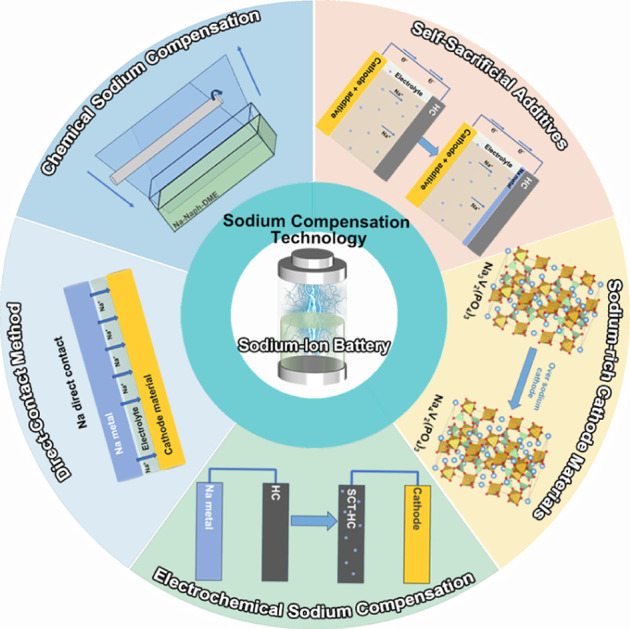

## 1 Introduction

Lithium-ion batteries (LIBs) play a dominant role in electrochemical energy storage,^1–4^ whereas energy storage systems require larger installed capacity, faster frequency regulation, and lower-cost batteries to support the rapid development of renewable energy sources.^5–8^ Moreover, the relatively low content of lithium in the Earth's crust (0.0017%), the high cost of extraction and refining, and its uneven distribution are the main factors limiting the application of lithium batteries for energy storage at scale. Hence, it is necessary to find lower cost alternatives to lithium.^9–13^ Sodium-ion batteries (SIBs) are a promising alternative due to the abundant raw materials and cost advantages.^14,15^ Besides, SIBs can use aluminum foil as the current collector for both electrodes due to the absence of the alloying reaction between Na and Al, which further reduces the overall costs.^16^ Moreover, SIBs exhibit greater stability at high temperatures and are less sensitive to temperature changes compared to their lithium-ion counterparts (LIBs). Research also shows that Na^+^ has a lower solvation energy, a faster interface ion diffusion capability, a higher ionic conductivity, and good safety.^16,17^ These attributes enable SIBs to achieve long-life performance across a wide temperature range, making them suitable for large-scale energy storage systems such as power grids and renewable energy storage.^18–20^ However, several technical challenges must still be addressed for widespread commercialization. The relatively poor electrochemical performance of SIBs compared to LIBs is not only attributed to the physical and chemical properties of electrode materials but also to the formation of a solid-electrolyte interphase (SEI) by the irreversible decomposition of the electrolyte as well as to a number of side reactions at the anode side, inducing a low initial coulombic efficiency (ICE).^21,22^ The cathode acts as the sole source of active Na^+^ ions for transport between the cathode and anode. The SEI formation process is also accompanied by the depletion of active sodium and irreversible capacity loss. Up to 15% of the active sodium ions will be inevitably consumed during the initial cycle, which may decrease the battery energy density by 20%.^23^ Therefore, it is of great significance to improve the ICE and maximize the performance of SIBs.

**Fig. 1:**
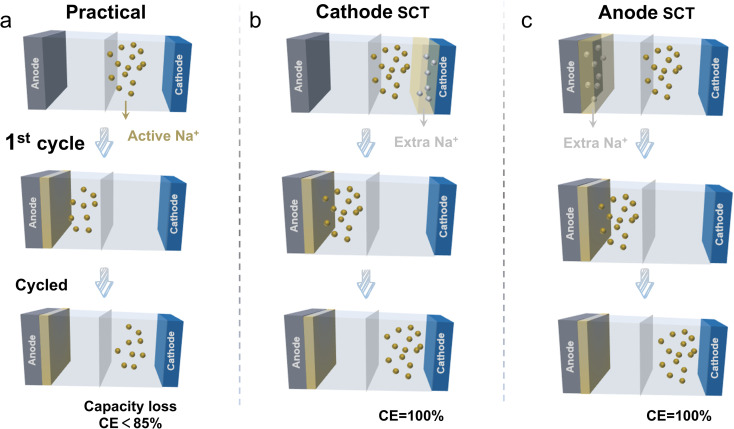
Schematic representation of the (de)-intercalation of sodium ions under (a) practical conditions; (b) the conditions with cathode sodium compensation; (c) the conditions with anode sodium compensation.

In order to effectively improve the ICE, researchers have proposed some methods to this end, mainly including: (i) reducing the consumption of sodium ions by decreasing the defects in the material;^24^ (ii) reducing the specific surface area of the material to decrease the consumption of the electrolyte;^24^ (iii) adding film-forming additives^22,24^ to the electrolyte that are beneficial for stabilizing the SEI film; and (iv) using the sodium compensation technique (SCT) to donate additional Na^+^ to compensate for the irreversible loss of Na^+^ during the cycling process.^25,26^ However, the effect of material optimization is relatively limited for reaching a high ICE of >90%.^27^ It is also difficult to accurately form a favorable SEI layer by suitable film-forming electrolyte additives. Meanwhile, the method is not widely used due to the lack of suitable additive species. In contrast, SCT is an effective means to boost the ICE by introducing additional sodium ions, which has the prospect of large-scale industrialization to realize a 100% ICE. As displayed in Fig. 1, in comparison to the practical conditions, using the sodium compensation technique (SCT) to donate additional Na^+^ to compensate for the irreversible loss of Na^+^ during the cycling process realizes a 100% ICE. Upon battery operation, the SCT-treated anodes showed a flattened and decreased potential profile, indicating a higher energy density and wider voltage range for the full cell.^28^ The SCT treatment on cathodes is able to supplement the Na^+^ loss especially for the sodium-deficient cathode (*e.g.*, P2-phase oxide, Na_2/3_Ni_1/3_Mn_2/3_O_2_),^29^ which helps to stabilize the electrolyte concentration and cathode material structure. This method of supplementing the loss of Na^+^ plays a crucial role in stabilizing the material structure and extending the battery life.^18,22^ SCT for the initial sodium loss by introducing additional sodium ions into the system can ensure a sufficient concentration of Na^+^ ions in the electrolyte right from the beginning. This process not only helps maintain the overall electrochemical balance but also effectively mitigates irreversible sodium loss. As a result, it preserves the structural integrity of the electrode, enhances the capacity and stability of the active material, and promotes more stable battery operation. Moreover, the uniform distribution of Na^+^ ions significantly reduces the likelihood of dendrite formation, thus minimizing the risk of battery failure.

**Scheme 1:**
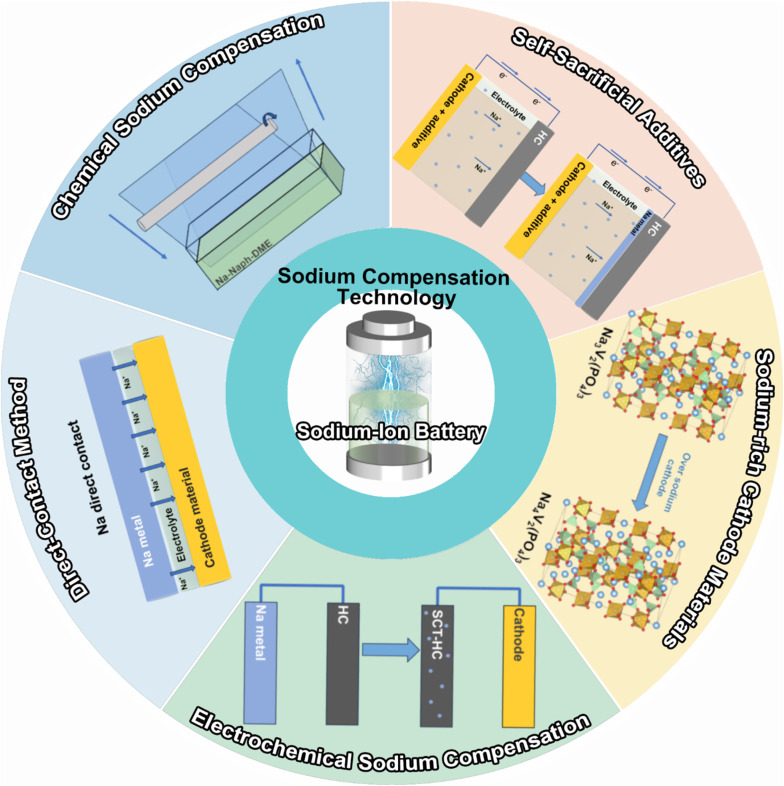
Outline of the most studied sodium compensation technologies.

SCT has emerged as the most straightforward and efficient way to address the cathode and anode material state matching issue by boosting energy density and offsetting active sodium-ion loss, two factors that are essential for the practical use of SIBs. By adding extra active sodium ions to the electrode material beforehand, the goal is to completely eradicate the permanent capacity loss upon the initial cycle. This work provides a timely extensive overview of the most recent advancements in the field of SCTs, including a chemical method using a Na complex, electrochemical approach, short circuit with sodium metal, cathode sacrificial additives, and sodium-rich cathode materials (Scheme 1). The merits and demerits of current approaches are carefully examined and deliberated in terms of the reaction mechanism, safety, compatibility, effectiveness, and scalability. After the SCT treatment, the ICE of the battery can become ∼100%, which indicates that the energy density can be effectively improved. The two most promising approaches for commercial applications—chemical method and cathode sacrificial additives—are highlighted as state-of-the-art developments. We also explore the unsolved scientific issues and technical challenges from a practical perspective. This timely review may encourage continued development of high-energy SIBs and offer recommendations for the study of cutting-edge sodium compensation technology.

## 2 Sodium compensation technology

### 2.1. Direct contact method

**Fig. 2:**
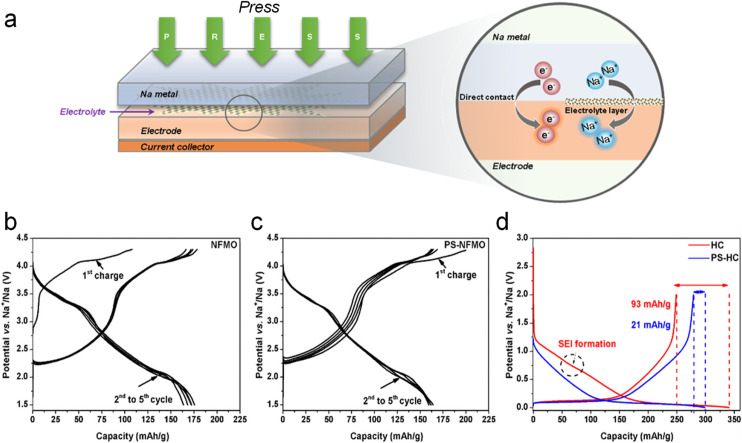
(a) Schematic illustration of direct contact SCT. Charge–discharge profiles of Na_0.67_Fe_0.5_Mn_0.5_O_2_ (b) before and (c) after sodium compensation. (d) Galvanostatic charge–discharge curves of HC electrodes with/without SCT. Reproduced with permission.^30^ Copyright 2019, American Chemical Society.

The direct contact sodium compensation method is performed by directly contacting sodium metal with an electrode (*e.g.*, hard carbon) using a small amount of electrolyte. To illustrate, sodium metal is placed in direct contact with the anode after adding a small amount of electrolyte (*e.g.*, 0.5 ml) and applying external pressure for a certain period (*e.g.*, 30 min). Using the large potential difference (*e.g.*, 2.7 V) between the anode (hard carbon) and the sodium metal, sodium metal is converted into sodium ions by losing electrons. These sodium ions are subsequently driven into the anode by a current that compensates for the depletion of active sodium within it as displayed in Fig. 2a. This process can occur at room temperature or elevated temperatures, depending on the materials and the desired rate of reaction. The sodium ions migrate into the electrode materials, occupying sites within the structure that will later accommodate sodium during battery operation. And they can react with the electrode materials forming sodium compounds (*e.g.*, Na_2_Ti_3_O_7_ from Ti_3_O_7_) that are stable within the electrode matrix. These compounds act as sodium reservoirs, providing sodium ions upon battery operation. This reaction is hinged on the electrochemical self-discharge principle, and the extent of sodium compensation can be roughly controlled by adjusting the length of the contact time.

Moeez *et al.*^30^ proposed the use of Na metal in direct contact with a hard carbon (HC) anode and a sodium-deficient P2-type Na_0.67_Fe_0.5_Mn_0.5_O_2_ cathode, respectively, which not only succeeded in compensating for the sodium loss on the anode side, but also provided the cathode with more active sodium ions. It is found that a favorable passivating layer can be formed on the cathode surface to suppress material dissolution. As such, the initial charging capacity of the Na_0.67_Fe_0.5_Mn_0.5_O_2_ cathode (Fig. 2b) was increased from 109 mA h g^−1^ to 200 mA h g^−1^ (SCT-treated Na_0.79_Fe_0.5_Mn_0.5_O_2_; Fig. 2c); the ICE of the HC electrode was improved from 73% to 94% with a significant reduction of the irreversible capacity loss by 70 mA h g^−1^ (Fig. 2d).

It shows that the direct contact method can pre-introduce active sodium ions into the electrode material by a facile and effective process. However, it is difficult to accurately control the SCT extent of the electrode material. This is due to the fact that for electrode materials, when the SCT extent is high, sodium can be easily precipitated at the anode side, resulting in safety hazards; a low SCT extent, nevertheless, is insufficient to make up for the loss of active sodium.

### 2.2. Electrochemical sodium compensation

Electrochemical sodium compensation is reported to accurately adjust the extent of SCT by controlling the current density and cut-off voltage of the half cell. The prepared electrode (*e.g.*, HC) is pre-cycled against a metallic sodium counter electrode to drive sodium ions into the electrode material by an external voltage, followed by removing the SCT-treated electrode for further full-cell assembly.

**Fig. 3:**
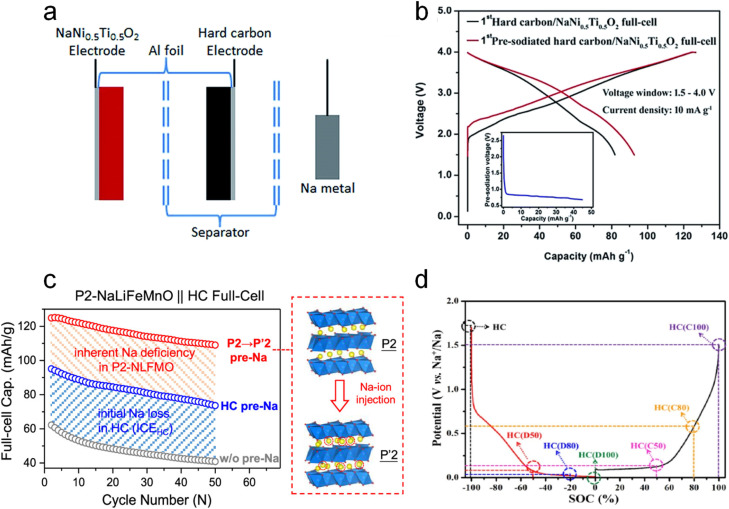
Electrochemical sodium compensation method. (a) Schematic diagram of a three-electrode configuration toward the sodium compensation of a hard carbon anode and (b) the initial charge–discharge curve of the full cell with the NaNi_0.5_Ti_0.5_O_2_ cathode and HC anodes with/without SCT. Reproduced with permission.^31^ Copyright 2015, the Royal Society of Chemistry. (c) Schematic diagram of phase transformation of P2-Na_0.67_Li_0.1_Fe_0.37_Mn_0.53_O_2_ to P′2-type Na_0.99_Li_0.1_Fe_0.37_Mn_0.53_O_2_ after SCT treatment and corresponding cycling performance comparison. Reproduced with permission.^26^ Copyright 2024, American Chemical Society. (d) Seven HC electrodes with different SOCs and corresponding potentials. Reproduced with permission.^32^ Copyright 2021, Elsevier.

Dating back to 2015, Wang *et al.*^31^ pioneeringly reported this SCT on the hard carbon anode in a three-electrode configuration (HC serves as the working electrode, and Na foil is the reference/counter electrode) under 10 mA g^−1^ as shown in Fig. 3a. Compared with the pristine HC‖NaNi_0.5_Ti_0.5_O_2_ full cell, the ICE of the SCT treated one increased from 64.5% to 73.0% (Fig. 3b). It also presented better cycling stability with 72.0% capacity retention after 100 cycles, higher than the 69.5% of the pristine cell. Inspired by this work, loads of reports adopted this SCT to pre-introduce enough active sodium ions into HC anodes and to form a favorable SEI layer with Na metal before assembling full cells in order to accurately evaluate the real application potential of the as-prepared cathodes.^19,33–38^

Other than HC anodes, this SCT can be employed in cathodes as well. Notably, P2-type layered oxides hold great promise as cathode materials for SIBs due to the outstanding rate capability and cycling stability. The main obstacle for them lies in the intrinsic Na deficiency (*e.g.*, Na_2/3_Ni_1/3_Mn_2/3_O_2_), greatly limiting the capacity performance of the full cell since it is the only source of active sodium in the full-cell configuration. In this regard, Fang *et al.*^26^ very recently precisely injected 0.32 excess active sodium per chemical formula unit into P2-phase Na_0.67_Li_0.1_Fe_0.37_Mn_0.53_O_2_ to realize P′2-type Na_0.99_Li_0.1_Fe_0.37_Mn_0.53_O_2_ by using the electrochemical SCT (discharging the Na‖Na_0.67_Li_0.1_Fe_0.37_Mn_0.53_O_2_ half battery to 1.5 V). It is reported that this process significantly improved the specific capacity from 61 mA h g^−1^ to 125 mA h g^−1^ for full cells coupled with HC anodes. It also presented noticeably better cycling performance compared to not only the pristine full cell but also the full cell with an SCT-treated HC anode (Fig. 3c).

With the purpose of disclosing the relationship between the sodium compensation degree of electrodes and full-cell performance, Pi *et al.*^32^ performed this SCT on the HC electrode with Na metal in half cells and subjected them to a certain state of charge (SOC) at a constant current rate of ∼0.05C (Fig. 3d), followed by removing the SCT treated HC and coupling it with an Na_3_V_2_(PO_4_)_2_F_3_ cathode to build a full cell. It is found that the electrochemical behavior of the HC‖Na_3_V_2_(PO_4_)_2_F_3_ full cell is highly dependent on different SOCs (*i.e.*, sodium compensation degree). The 0% SOC (fully discharged) HC could enable an optimal electrochemical performance: an initial discharge capacity of 114.1 mA h g^−1^ and a capacity retention of 71.8% throughout 600 cycles at 10C. This HC is able to offer sufficient active sodium for compensating for the loss of active sodium from SEI generation and irreversible intercalated sodium sites. This work sufficiently proves the advantage of electrochemical SCT in controlling the extent of sodium compensation.

Thus, electrochemical sodium compensation is an effective approach to improve the ICE of the electrode by modulating the external circuitry to precisely adjust the SCT degree and regulate the rate of SEI film formation, thus improving the performance of the subsequent full cell. In comparison to other SCTs, it may also maximally preserve the actual state of sodiation in the first cycle without causing additional undesirable reactions, which has the least detrimental impact on battery performance. However, half-cell disassembly and reassembly of the full cell greatly increase the process complexity and difficulty. In addition, the residual sodium metal in the process has high reactivity and is difficult to handle, increasing the overall cost and limiting its practical application.

### 2.3. Physical sodium compensation of the sodium-based metal

In comparison to the above two SCTs, the potential for large-scale application of sodium metal physical SCT is higher. This is attributed to the high theoretical capacity of sodium (1166 mA h g^−1^) and low redox potential (−2.741 V *versus* standard hydrogen electrode), which can be directly physically compounded with the electrode active material by using a small amount of sodium metallic powder or sodium foil to quantitatively and controllably pretreat the electrode with active sodium. Several sodium metal physical SCTs have been developed.

**Fig. 4:**
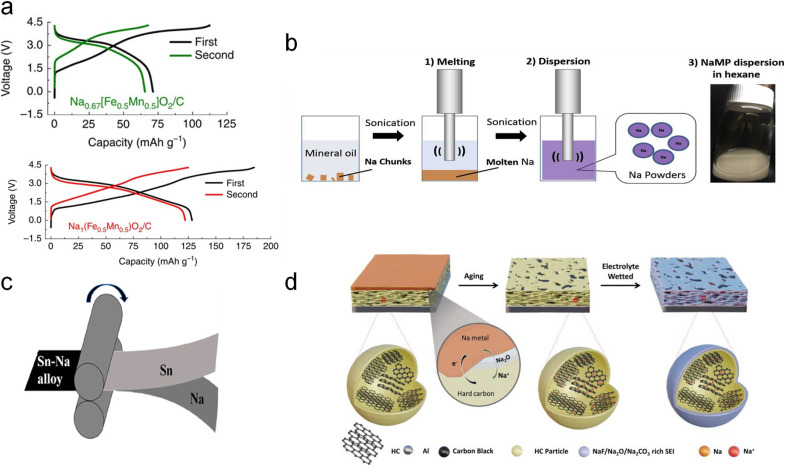
(a) Charge–discharge profiles of Na_0.67_[Fe_0.5_Mn_0.5_]O_2_ and Na_1_[Fe_0.5_Mn_0.5_]O_2_ by ball milling with Na metal. Reproduced with permission.^39^ Copyright 2016, Springer Nature. (b) Pulsed ultrasonic SCT dispersion method. Reproduced with permission.^40^ Copyright 2018, Elsevier. (c) Diagrammatic illustration of the metallurgical pre-alloying process of Sn–Na. Reproduced with permission.^41^ Copyright 2019, American Chemical Society. (d) Schematic illustration of solid-state electrochemical SCT on the HC electrode. Reproduced with permission.^42^ Copyright 2024, Wiley-VCH.

In 2016, Zhang *et al.*^39^ for the first time reported a facile and scalable ball milling method on Sb and P powders to prepare various Na-based alloys such as Na_3_Sb and Na_3_P as the sacrificial sodium source with high initial charging capacities (Fig. 4a). Specifically, Na_3_P and Na_3_Sb powders were obtained through a solid-state reaction by ball milling stoichiometric amounts of bulk metallic sodium red phosphorus (P) or antimony (Sb) powders respectively in an argon atmosphere to prevent oxidation of sodium for 2 hours based on a ball-to-material mass ratio of 35. The underlying mechanism can be the solid-state alloying reaction, which allows sodium atoms to diffuse into the lattice of P or Sb, forming Na_3_P and Na_3_Sb alloys respectively under high-energy ball-milling conditions. These compounds are formed through direct combination reactions: 3Na + P → Na_3_P; 3Na + Sb → Na_3_Sb. In addition, this approach is also suitable for sodium compensation of a sodium-deficient insertion layered oxide (Na_0.67_Fe_0.5_Mn_0.5_O_2_) and polyanion compound (Na_3_V_2_(PO_4_)_2_F_3_) to build pure Na_1_Fe_0.5_Mn_0.5_O_2_ and Na_4_V_2_(PO_4_)_2_F_3_ phases with a noticeable capacity improvement. The as-prepared Na-rich cathode materials effectively make up for sodium loss from the SEI formation process and thus enable a substantial increase in energy density of full cells when coupled with HC anodes. The HC‖Na_1_Fe_0.5_Mn_0.5_O_2_ full cell presented an increased discharge capacity from 71 mA h g^−1^ to 128 mA h g^−1^, and the energy density was boosted by 30% compared to the pristine one.

Additionally, Tang *et al.*^40^ proposed an ultrasonic dispersion method for SCT on HC anodes (Fig. 4b). Specifically, Na chunks in mineral oil were melted at 98 °C and ultrasonically treated to form a hexane-based Na powder suspension (2–16 μm in size) in an inert atmosphere, followed by a spin coating process on the HC anode surface. It decreases irreversible ICE to 8% from the initial 19.3%. ∼5% increment in energy density of the HC‖NaCrO_2_ full cell is thus realized.

Furthermore, Liu *et al.*^43^ performed SCT on a Sn anode using a roll-to-roll process to metallurgically generate Na–Sn alloys *in situ* from sodium metal foil and Sn anodes (Fig. 4c). To perform the metallurgical pre-alloying process of sodium, Na foil was overlayed on a Sn foil and the two foils were rolled to form a Na–Sn alloy. This is based on the solid-state alloying reaction Na + Sn → Na–Sn. It has been found that when pure Sn foil was used as the anode, Sn easily catalyzed the rapid decomposition of the electrolyte to produce large amounts of CO_2_ and H_2_ gases at a specific voltage. An excessively thick SEI film on the electrode surface deactivated the Sn electrode accordingly. In contrast, the as-formed Sn–Na alloy lowers the open-circuit potential, thus inhibiting the gas-related side reactions. Thus, the ICE of the Sn–Na‖Na_3_V_2_(PO_4_)_2_F_3_ full cell increased significantly from 24.68% to 75%. In addition, the Na–Sn alloy presents a good air stability after exposure to air for 2 h. It has good potential for large-scale application considering the operability of the roll-to-roll process.

Wang *et al.*^42^ very recently reported a new vacuum thermal evaporation SCT method on HC anodes by controlling the thickness of deposited sodium metal to 1 and 2.3 μm from a quartz crystal microbalance, followed by being aged for 3–5 days under an Ar atmosphere (Fig. 4d). The chemical potential difference between Na metal and HC drives the sodium compensation process and brings about a thin Na_2_O SEI layer for fast Na^+^ transport. The full cell with the Na_3_V_2_(PO_4_)_3_ cathode presents an improved capacity (from 66.1 mA h g^−1^ to 102.2 mA h g^−1^), a boosted ICE (from 61% to 94%), and enhanced capacity retention. Besides, the as-prepared HC displays good air stability. This technology can accurately adjust the SCT degree and avoids direct usage of metallic sodium, which should be paid more attention in practical application.

Although sodium-based metal physical SCT is simple, the residual sodium metal will easily turn into “dead sodium”, leading to an increase in the polarization of the battery and sodium precipitation phenomenon. And sodium powder or sodium foil has technical difficulties (soft texture) and safety risks (high reactivity; high sensitivity to water and air) during production, transportation and use. How to improve their air stability is the key to realize their large-scale application.

### 2.4. Chemical sodium compensation by a Na complex

The high reactivity and risky properties of metallic sodium should be kept in mind. Instead, chemical sodium compensation by a Na complex is considered one of the most promising SCTs by virtue of its safety, practicality, and ease of industrialization, and thus has received increasing attention in recent years. This is achieved by employing the large potential difference between the chemical sodiation reagent and electrode material. Chemical sodiation reagents are usually generated *in situ* from sodium metal and aromatic compounds in organic solvents. Commonly used aromatic compounds include biphenyl (Biph), naphthalene (Naph), *etc.*, and the organic solvents usually are ethylene glycol dimethyl ether (DME), tetrahydrofuran (THF), *etc*. In the process, the electrons and sodium ions of the chemical sodiation reagent will be automatically transferred to the electrode material (*e.g.*, HC) for sodium compensation within a short period due to the strong reducing capability of the chemical sodiation reagent. Accurate control of the SCT depth (ICE can reach ∼100%) can be attained by controlling the reaction time and temperature as well as the variety and concentration of the sodiation reagents.

In 2019, Cao *et al.*^44^ performed chemical SCT on a Na_2_Ti_6_O_13_ anode by immersing it into sodium naphthalene solutions (Na-Naph-DME) at different concentrations. The Na-Naph@DME solution was obtained by adding naphthalene to ethylene glycol dimethyl ether (DME) with the same number of moles of metallic sodium. Na_2_Ti_6_O_13_ was then put into the above solution for sodium compensation. The ICE of the pretreated Na_2_Ti_6_O_13_‖Na_3_V_2_(PO_4_)_3_ full cell was increased from 40% to 80%.

**Fig. 5:**
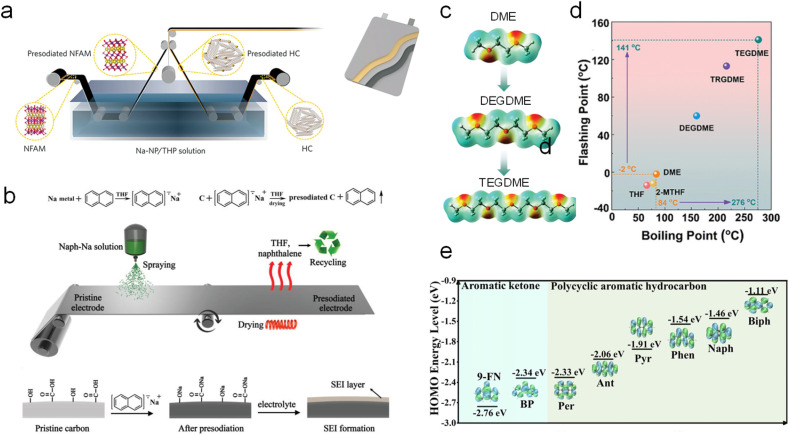
(a) Schematic of the Na–NP@THP solution-based chemical sodium compensation method for a commercial roll-to-roll process. Reproduced with permission.^45^ Copyright 2023, Wiley-VCH. (b) Illustration of spraying the Na–Naph@THF solution manufacturing process. Reproduced with permission.^46^ Copyright 2019, Wiley-VCH. (c) Electrostatic potential mapping and (d) boiling and flashing points of various solvents. Reproduced with permission.^47^ Copyright 2024, Wiley-VCH. (e) HOMO energy levels of anionic radicals of PAHs and aromatic ketone. Reproduced with permission.^48^ Copyright 2024, Wiley-VCH.

Afterwards, Wang *et al.*^45^ reported a roll-to-roll chemical SCT by simultaneously immersing a P2-type Na_0.67_Fe_0.1_Al_0.1_Mn_0.8_O_2_ cathode and HC anode in a sodium (Na)–naphthalene (NP)@tetrahydropyran (THP) solution for a certain period (Fig. 5a). This strategy builds favorable interphases around both electrodes, which not only facilitate interfacial charge transfer but also mitigate notorious side reactions (*e.g.*, transition metal dissolution, electrolyte decomposition, and destruction of the CEI/SEI). As such, the HC‖Na_0.67_Fe_0.1_Al_0.1_Mn_0.8_O_2_ pouch cell presented excellent capacity retention of 93.4% throughout 100 cycles. However, it is still faced with the problems that the immersion process requires a large amount of SCT reagent and the residual reagent on the electrode surface is difficult to be completely removed.

In this regard, Sun *et al.*^46^ reported a simple but efficient spraying approach. Sodium metal and naphthalene (Naph) with an equal molar amount were added into tetrahydrofuran (THF) to form Na–Naph@THF solution. And then a certain amount of this strongly reductive solution was directly sprayed onto the HC surface for sodium compensation. During this treatment, the residual reagent (*i.e.*, naphthalene and tetrahydrofuran) was evaporated for recycling to reduce the impact on the battery (Fig. 5b). The entire process not only achieves sodium compensation, but also recycles organic matter during the drying process, which greatly reduces the process cost. The ICE of the SCT-treated HC was increased from 67% to 87%. The energy density of the full cell with a Na_0.9_[Cu_0.22_Fe_0.30_Mn_0.48_]O_2_ cathode was increased from 141 W h kg^−1^ to 240 W h kg^−1^.

Nevertheless, the redox potential of Na-Naph@THF is high (∼0.2 V *versus* Na^+^/Na) and can only partially SCT-treat the electrode, impossibly achieving a 100% ICE. Alternatively, a sodium compensation reagent with a lower desodiation potential of 0.12 V (Na–Bp@DME; Bp = biphenyl) was developed by Liu *et al.*^49^ The ICE of HC thus achieved 100%, and a uniform and dense SEI film was formed on the surface, which is conducive to the improvement of its air stability. The energy density of the HC‖Na_3_V_2_(PO_4_)_2_ full cell was increased from 120 W h kg^−1^ to 218 W h kg^−1^. Afterwards, Li *et al.*^50^ reported a Na–Bp@THF solution presenting a lower desodiation potential of 0.095 V to effectively improve the ICE of Sn and P anodes. Qin *et al.*^51^ further developed an *in situ* electrochemical SCT *via* introducing Na–Bp@DME reagent as an electrolyte additive into a DME-based ether electrolyte. It realizes sodium compensation on HC anodes with a ∼100% ICE due to the theoretical calculation result that the lowest unoccupied molecular orbital (LUMO) energy level of HC resembles the highest occupied molecular orbital (HOMO) energy level of Na–Bp reagent and their symmetry matches well.

However, an extremely low redox potential of polycyclic aromatic hydrocarbons (PAHs) will cause the electrode structure to deteriorate and incur sodium accumulation with safety hazards. Zhang *et al.*^48^ very recently designed mild chemical SCT reagents' molecular structure *via* introducing a carbonyl group in PAHs to balance the conjugated and inductive effect. Benzophenone (BP) and 9-fluorenoneb (9-FN) stand out as the optimal candidates with favorable redox potentials of 1.07 V and 1.55 V (*versus* Na^+^/Na), respectively (Fig. 5e). 9-FN manifests versatile sodium compensating abilities for various cathode materials including layered Na_0.67_Ni_0.33_Mn_0.67_O_2_, tunneled Na_0.44_MnO_2_, polyanionic Na_3_V_2_(PO_4_)_2_F_3_, Na_3_V_2_(PO_4_)_3_, and Na_4_Fe_2,91_(PO_4_)_2_P_2_O_7_ with greatly boosted initial charging capacity to compensate for sodium loss in the anode side. Fang *et al.*^28^ also reported a sodium diphenyl ketone in dimethyl ether (Na-DK@DME) reagent featuring a redox potential of 0.55 V for sodium compensation of HC anodes with a greatly boosted ICE of 99.2%.

On the other hand, practical application is hindered by the high reactivity of presodiated electrodes. Also, there are safety issues for widespread use due to the volatility of the toxic aromatic reagents, low flash point and boiling point of the commonly used ether solvents, and the use of sodium metal in the production of sodium-containing aromatic radicals, which deserves more attention. Thus, Gao *et al.*^53^ applied sodium bis(2-methoxyethoxy) aluminum hydride (*i.e.*, NaAlH_2_(OCH_2_CH_2_OCH_3_)_2_) for sodium compensation on HC anodes with a high ICE of 94.6%, and they found that a passivated Al_2_O_3_ nanolayer was *in situ* built to endow SCT-treated HC anodes with good air stability. Man *et al.*^47^ molecularly designed linear ether solvent and screened out tetraethylene glycol dimethyl ether (TEGDME) as an optimal candidate due to its high boiling point of 276 °C and flash point of 141 °C (*versus* 84 and −2 °C for DME) to intrinsically enhance the safety (Fig. 5c and d). The designed sodium 4-methylbiphenyl (4-MBP) in TEGDME (*i.e.*, Na-4-MBP@TEGDME) SCT reagent greatly improved the ICE of HC from 65.28% to 99.1%.

In general, chemical sodium compensation can be controlled by adjusting the concentration of the Na complex solution and the reaction time. The danger associated with the strong activity of Na metal can be minimized. The spraying of the Na-complex solution on the electrode surface facilitates efficient and homogeneous sodium compensation compared to the other methods mentioned above, and the roll-to-roll process helps in the mass production of sodiated electrodes. In addition, sodium compensation reagents can be used for various types of electrode materials (Table 1), and the recycling of organic reagents through process optimization can significantly reduce costs. However, the air instability of treated electrodes remains to be solved.

**Table 1:** Comparison of different electrodes before and after chemical SCT^a^

Electrode	Sodium compensation reagent	ICE of the half-cell before/after SCT	ICE of the full cell after sodium compensation	Ref.
Na_2_Ti_6_O_13_	Na–Naph@DME	65%/100%	80.0% (Na_2_Ti_6_O_13_‖Na_3_V_2_(PO_4_)_3_)	44
Na_2_Ti_3_O_7_		∼43%/∼100%	None	44
TiO_2_		∼75%/∼100%	None	44
Hard carbon	Na–Naph@THF	67%/87%	92.9% (HC‖Na_0.9_[Cu_0.22_Fe_0.30_Mn_0.48_]O_2_)	46
Sb	Na–Biph@DME	75%/∼100%	95.4% (Sb‖Na_3_V_2_(PO_4_)_3_)	52
Sn	Na–Biph/THF	70%/94%	None	50
P/C		64%/94%	None	50

a Naph (sodium naphthaline); DME (methoxymethane); THF (tetrahydrofuran); NaBiph (sodium biphenyl).

Different methods discussed above possess unique strengths and weaknesses. The direct contact method and the sodium-metal physical mixing approach offer advantages such as easy control, a high sodium compensation capacity, and a sufficiently low redox potential. However, their reliance on sodium metal in the preparation process presents significant safety hazards. The high reactivity of sodium metal complicates precise control of sodium compensation in the direct contact method, leading to non-uniform SEI film formation and potential material degradation. To achieve a uniform SEI film, the electrochemical sodium compensation method controls sodium compensation precisely by assembling a half-cell to regulate current density and charging time. Nevertheless, the method's complexity, involving the assembly and disassembly of half-cells, hinders its scalability. Moreover, residual metallic sodium after disassembly remains a safety concern.

In contrast, the chemical sodium compensation by a Na complex method offers simplicity and easy control of the SCT degree by adjusting reaction time. By utilizing sodium compounds instead of sodium metal, it avoids the safety hazards associated with metallic sodium. However, challenges persist such as sensitivity of sodium compounds to air and insufficient redox potential, which require resolution for optimal performance.

In summary, each sodium compensation method for SEI film formation presents trade-offs in terms of control, safety, and technical feasibility. The choice of method depends on specific application requirements, including safety considerations, scalability, and desired SEI film characteristics. Continued research and development are essential to address these challenges and advance battery technology effectively.

The above sodium compensation methods use sodium metal as a sodium source to provide Na^+^ ions or to form a strongly reducing sodium compensation reagent, which incurs safety hazards. In contrast, sodium compensation additives applying cathode materials are a promising and relatively safer strategy for compensating for the initial active sodium loss. It achieves sodium compensation by an extra amount of active sodium ions during the initial charging process and it can be categorized as self-sacrificial additives and sodium-rich cathode materials.

### 2.5. Self-sacrificial additives

The ideal sodium compensation additives should feature the following characteristics: (i) high sodium compensation capacity (>350 mA h g^−1^) to provide sufficient active sodium ions; (ii) appropriate decomposition potential for irreversible desodiation within the operating voltage range of the cathode; (iii) the additive itself and oxidative by-product have no impact on the subsequent cycling of the battery; (iv) technological compatibility with current processes. The performance evaluation of sodium compensation additives mainly focuses on these four perspectives. There are many types of additives for the cathode, which can be roughly divided into two categories: inorganic and organic.

#### 2.5.1. Inorganic sodium compensation additives

**Fig. 6:**
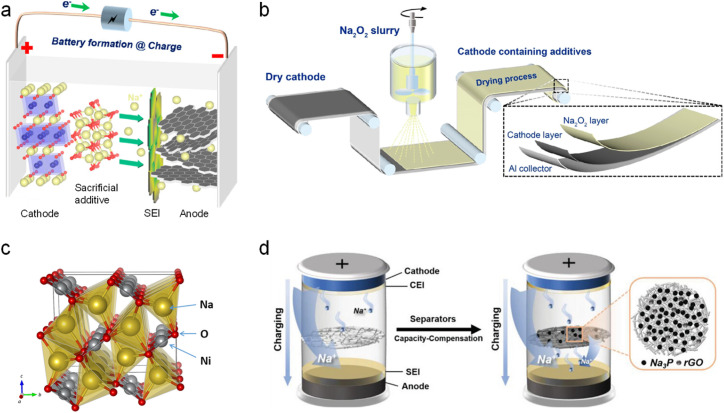
(a) Schematic illustration of the self-sacrificial additive strategy. (b) Schematic diagram of electrodes prepared by spraying a slurry containing Na_2_O_2_. Reproduced with permission.^56^ Copyright 2021, American Chemical Society. (c) Crystal structure of Na_2_NiO_2_. Reproduced with permission.^57^ Copyright 2015, American Chemical Society. (d) Schematic illustration of a separator coated by Na_3_P@rGO sodium compensation additives. Reproduced with permission.^58^ Copyright 2023, Wiley-VCH.

Dating back to 2013, NaN_3_ was first reported as a sodium compensation additive by Singh *et al.*^54^ to provide active sodium (2NaN_3_ → 2Na^+^ + 3N_2_↑ + 2e^−^) with a high theoretical capacity of 412 mA h g^−1^ and a favorable decomposition potential of 3.55 V (*versus* Na^+^/Na). Afterwards, IIarduya *et al.*^55^ applied this additive (20 wt%) in P2-type Na_0.67_Fe_0.5_Mn_0.5_O_2_ with increased initial charging capacity from 50 mA h g^−1^ to 130 mA h g^−1^, as shown in Fig. 6a. Nevertheless, the large amount of N_2_ gas produced by the decomposition of NaN_3_ and explosive properties of NaN_3_ limit its large-scale application.

Many other SCT additives similar to NaN_3_ were subsequently reported such as NaBH_4_,^59^ NaNH_2_,^60^ Na_2_O,^61^ Na_2_O_2_,^56^ Na_2_CO_3_,^62^ and NaNO_2_.^63^ Gas evolution, however, remains as a main obstacle and will lead to the formation of pores on the surface of the electrode, which is not conducive to the structural stability of the electrode and battery safety (H_2_ gas from NaBH_4_ or NaNH_2_). In this regard, Guo *et al.*^56^ developed a spraying process by spraying a slurry of acetonitrile solution containing Na_2_O_2_ additives to form a Na_2_O_2_ sodium compensation layer onto the surface of the dry Na_2/3_Ni_1/3_Mn_1/3_Ti_1/3_O_2_ cathode (Fig. 6b). Upon charging, this SCT layer will decompose at 4 V (*versus* Na^+^/Na; Na_2_O_2_ → 2Na^+^ + O_2_↑ + 2e^−^) to provide active sodium ions (charging capacity: 421.5 mA h g^−1^) and more importantly, avoids the porous structure of the underlying electrode by O_2_ gas evolution. Otherwise, O_2_ gas was reported to react with the SCT-treated electrode vigorously to deplete the active sodium ions of the battery, which accounts for the low utilization extent of this type of additive. The initial discharge capacity of the HC‖Na_2/3_Ni_1/3_Mn_1/3_Ti_1/3_O_2_ with 20% Na_2_O_2_ was 81.7 mA h g^−1^, and the capacity retention was 84.1% after 80 cycles at 0.5C, which were significantly higher than those of the pristine full cell (60.3 mA h g^−1^ and 61.3%).

To avoid the negative effect of the gas byproduct on the electrode, some other inorganic sodium compensation additives including Na_3_P, Na_2_S, Na_2_NiO_2_, *etc*. without generating gas entered the researchers' field of vision. Park *et al.*^57^ in 2015 applied orthorhombic Na_2_NiO_2_ (Fig. 6c) as a cathode SCT additive (Na_2_NiO_2_ → NaNiO_2_ + Na^+^ + e^−^, at 2.1 V *versus* Na^+^/Na) with a charging capacity of 285 mA h g^−1^. Only 10 wt% Na_2_NiO_2_ additive into the NaCrO_2_ cathode is able to increase the reversible capacity of the Sb/C‖NaCrO_2_ full cell from 74.6 mA h g^−1^ to 92.1 mA h g^−1^. However, the resultant crystalline NaNiO_2_ can serve as a cathode active species showing a low reversible capacity of 88.7 mA h g^−1^ in the subsequent cycles, which greatly reduced the sodium replenishment effect of the additive.

Considering the high capacity of 804 mA h g^−1^ of Na_3_P, Mao *et al.*^58^ reported a separator capacity-compensation strategy by combining Na_3_P with a conductive rGO coating layer, which was located on the side of the separator facing the cathode (Fig. 6d). It is found that Na_3_P starts to desodiate at 4.05 V *versus* Na^+^/Na with an actual capacity of 478 mA h g^−1^. The initial specific capacity of the HC‖NaNi_1/3_Fe_1/3_Mn_1/3_O_2_ full cell with the Na_3_P@rGO-coated separator was increased to 128.2 mA h g^−1^ from 108.5 mA h g^−1^ (*i.e.*, 18.2%). This strategy can be also applied in the HC‖Na_3_V_2_(PO_4_)_3_ full cell with a capacity increase of 36.2%. Furthermore, they identified that the remaining phosphorus after the decomposition of Na_3_P could prevent oxygen evolution from the cathode at high voltages due to its high reactivity with active oxygen species, thus boosting battery safety. However, Na_3_P has high reactivity to air, and is easily converted into highly toxic PH_3_, which impede its use.

Comparatively, Na_2_S seems to be better than Na_3_P as an alternative sodium compensating additive, effectively avoiding the generation of toxic gases. The sodium compensation capacity of Na_2_S is quite close to the theoretical capacity of 687 mA h g^−1^.^64^ Another candidate is Na_2_S. However, the decomposition of Na_2_S produces electronically and ionically insulated sulfur and polysulfides, which will increase battery resistance. Besides, polysulfide dissolution in the electrolyte is unfavorable for subsequent cycling stability and self-discharge of the battery. To address the above problems, Hu *et al.*^65^ proposed to transform Na_2_SO_4_ into Na_2_S *in situ* encapsulated in a carbon matrix (Na_2_S-4) with a boosted electronic conductivity and smaller particle size than the commercial counterpart. As a sodium compensation additive, the Na_2_S/C composite was able to achieve an initial charging capacity of 407 mA h g^−1^ at 3.8 V *versus* Na^+^/Na. The addition of 10 wt% Na_2_S/C into the Na_3_V_2_(PO_4_)_3_ cathode endows the HC‖Na_3_V_2_(PO_4_)_3_ pouch cell with a capacity increase of 24.3% and with an increment in the energy density from 117.2 to 138.6 W h kg^−1^. Moreover, the Na_2_S additive doesn't produce gas upon charging and its desodiated product has no negative influence on the subsequent cycling stability and rate capability of SIBs. Although the inorganic sodium compensation additive can effectively improve the energy density of the battery, there are still problems such as residual and gas formation after SCT treatment and high price, which restrict its large-scale production. Table 2 compares various types of inorganic SCT additives from different aspects.

**Table 2:** Comparison of key factors of inorganic cathode SCT additives

	Additive	Potential (V *versus* Na^+^/Na)	Theoretical/actual capacity (mA h g^−1^)	Main product	Problem	Ref.
Inorganic additive	NaN_3_	3.55	412/315	N_2_	Explosive	66
	Na_3_P	0.5	804/600	P	Toxic and flammable	39 and 65
	Na_2_O	2.5–4.2	864/500	O_2_	Production of H_2_	67
	Na_2_O_2_	4	687/421	O_2_	Production of O_2_	
	NaNO_2_	3.3–3.8	427/350	NO_2_	Production of NO_2_	63
	Na_2_CO_3_	4	506/253	CO_2_, O_2_	Production of O_2_	62
	Na_2_NiO_2_	2.0–3.6	392/277	NaNiO_2_	Limited sodium compensation	57
	NaCrO_2_	3.0–4.2	251/229	Na_0.06_CrO_2_	Limited sodium compensation	68
	NaBH_4_	2.4	708/750	B, H_2_	Production of H_2_	59
	NaNH_2_	3.8	686/680	N_2_H_4_, N_2_, H_2_	Production of H_2_ and N_2_H_4_ with strong reduction and instability	60
	Na_2_S	3.8	687/553	S	Low electronic conductivity	64

#### 2.5.2. Organic sodium compensation additives

Compared with inorganic sodium compensation additives, organic ones are widely available, cost-effective, and environmentally friendly. More importantly, their molecular structure is rich and diverse, and their decomposition potential and SCT capacity can be precisely controlled through molecule-scale structural design.

**Fig. 7:**
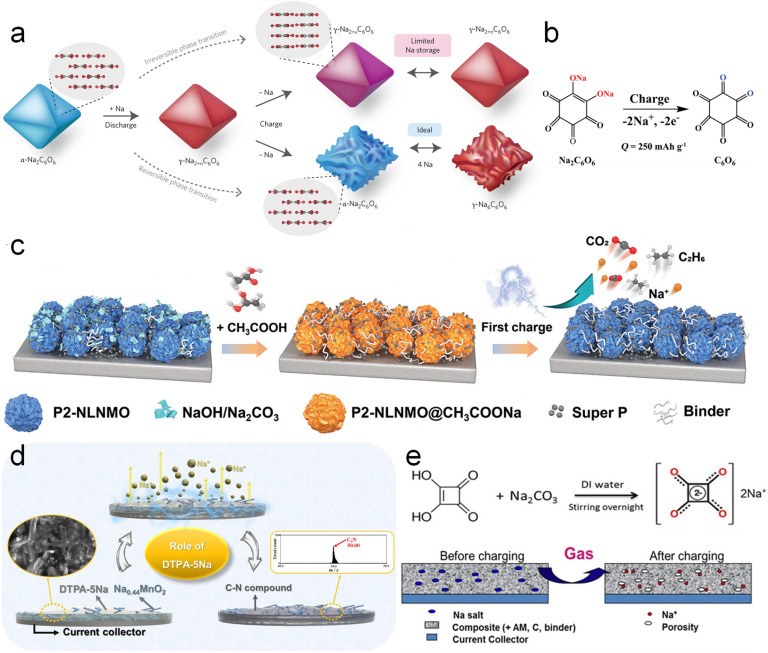
(a) A schematic of the sodium storage mechanism of Na_2_C_6_O_6_. Reproduced with permission.^69^ Copyright 2017, Springer Nature. (b) Scheme of the desodiation of Na_2_C_6_O_6_. Reproduced with permission.^70^ Copyright 2020, Wiley-VCH. (c) Schematic illustration of the Na compensation mechanism in P2-NLNMO@AC by acetic acid. Reproduced with permission.^71^ Copyright 2023, American Chemical Society. (d) DTPA-5Na sodium compensation schematic. Reproduced with permission.^72^ Copyright 2020, Elsevier. (e) Schematic diagram of sodium compensation concept by Na_2_C_4_O_4_. Reproduced with permission.^73^ Copyright 2018, Wiley-VCH.

Organic carbonyl compounds are usually electrochemically unstable at high voltages and undergo irreversible oxidative desodiation reactions and thus are widely used as sodium compensation additives. In 2017, Lee *et al.*^69^ for the first time discovered that the practical reversible capacity of disodium rhodizonate (Na_2_C_6_O_6_) is substantially lower than the theoretical specific value (501 mA h g^−1^). The irreversible phase transition of Na_2_C_6_O_6_ from the γ- to α-phase during the initial cycle accounts for the deterioration of its redox activity as displayed in Fig. 7a. Inspired by this, Zou *et al.*^70^ applied Na_2_C_6_O_6_ as a sodium compensation additive as shown in Fig. 7b. In typical carbonate-based battery electrolytes, Na_2_C_6_O_6_ with its stable sodium–oxygen bond is weakly soluble, but its desodiation product (cyclohexanehexone) is neutral and easily soluble, which may ensure the forward desodiation reaction. The actual charging capacity of Na_2_C_6_O_6_ was 309.8 mA h g^−1^ at 3.98 V *versus* Na^+^/Na, which is higher than the two-electron theoretical value of 250 mA h g^−1^ and indicates that there are some unknown side reactions. Thus, Marelli *et al.*^74^ employed online electrochemical mass spectroscopy, vibrational spectroscopy, and *in situ* XRD to further disclose that the products (rhodizonate) of Na_2_C_6_O_6_ desodiation catalyze the decomposition of the electrolyte or rhodizonate is further oxidized.

Recently, Zhang *et al.*^71^ neutralized surficial residual alkalis and thus formed sodium acetate (CH_3_COONa, AC-Na, Fig. 7c) by introducing acetic acid (AC) into layered cathode materials, which realized the goal of “turning the waste into wealth”. AC-Na, as a Na-compensated additive, has a high specific capacity of 300 mA h g^−1^ and an oxidation potential of 4.1 V *versus* Na^+^/Na. Based on sodium compensation, the 2 Ah-level HC‖Na_0.85_Li_0.12_Ni_0.22_Mn_0.66_O_2_@AC pouch cell presented a capacity retention of 95.1% over 120 cycles and achieved an energy density increase from 112 to 130 W h kg^−1^.

In response to possible electrolyte decomposition at such a relatively high oxidation voltage of 4.1 V, Zou *et al.*^75^ afterwards effectively lowered the oxidation voltage plateau of sodium carboxylate to 3.45 V *versus* Na^+^/Na by molecularly optimizing the conjugated aromatic architecture and introducing a stronger electron-donating substituent and thus sodium *para*-aminobenzoate (PABZ-Na) was proposed. Ethylenediaminetetraacetic acid tetrasodium salt (EDTA-4Na)^76^ and penta-sodium diethylenetriamine pentaacetate acid salt (DTPA-5Na)^72^ (Fig. 7d) have also been reported by Jo *et al.*^72^ as SCT additives to show charging capacities of 420 mA h g^−1^ at 3.79 V and 363 mA h g^−1^ at 3.6 V *versus* Na^+^/Na, respectively. But both of them produce H_2_O during the desodiation process and H_2_O will be further decomposed into H_2_ and O_2_, which will threaten battery safety and cycling stability.

Shanmukaraj *et al.*^73^ proposed Na_2_C_4_O_4_ as an SCT additive with a charging capacity of 256 mA h g^−1^ at 3.6 V *versus* Na^+^/Na (Na_2_C_4_O_4_ → 2CO_2_↑ + 2C + 2e^−^ + 2Na^+^) (Fig. 7e). The HC‖Na_3_(VOPO_4_)_2_F full cell with 30 wt% Na_2_C_4_O_4_ additives achieved an initial discharge capacity of 120 mA h g^−1^ at 0.05C, which is 50% higher than that of the unpretreated full cell (80 mA h g^−1^). A better battery performance was achieved compared to the NaN_3_-added cell, which was ascribed to the cathode porosity increase by the as-formed CO_2_. Thus, Pan *et al.*^77^ further investigated the decomposition process of Na_2_C_4_O_4_ by performing nitrogen adsorption, Raman spectroscopy and electrochemical mass spectrometry. A new decomposition mechanism of Na_2_C_4_O_4_ was identified: C_4_O_4_^2−^ ↔ 1.47CO_2_ + 1.06CO + 1.47C + 2e^−^. Upon charging, the generated CO was further partially proportionated into C and CO_2_ catalyzed by activated carbon in the electrode, which significantly improved the conductivity. Besides, the formed CO_2_ tends to react with the sodiation electrode to build the Na_2_CO_3_ passivating layer to effectively prolong battery lifespan.

For organic sodium compensation additives, a greater molecular weight means a lower theoretical sodium compensation capacity. Reducing the molecular weight from functional groups and increasing the proportion of sodium content is one of the effective ways to increase the sodium compensation capacity of additives. Niu *et al.*^78^ further designed and synthesized sodium oxalate (Na_2_C_2_O_4_) as a lightweight SCT additive with a low oxidation potential of 3.97 V *versus* Na^+^/Na and a high charging capacity of 394.6 mA h g^−1^. They also found that the incorporation of conductive carbon or noncarbon-based catalysts can effectively reduce the oxidation potential of sodium-compensated additives (from 4.41 to 3.97 V in this case). Similarly, Shanmukaraj *et al.*^79^ combined a sodium pyruvate (Na_2_C_3_O_4_) additive with KJ600 conductive carbon and then added it into a P2-Na_2/3_Mn_0.8_Fe_0.1_Ti_0.1_O_2_ cathode. The half-cell showed a discharge capacity of 164 mA h g^−1^ at 0.1C, which is 27% higher than that of the pristine one.

Table 3 compares various types of organic SCT additives. Therefore, it is foreseeable that molecular design of organic sodium compensation additives and more in-depth study of reaction mechanisms will be the future research direction.

**Table 3:** Comparison of key factors of organic cathode SCT additives

	Additive	Potential (V *versus* Na^+^/Na)	Theoretical/actual capacity (mA h g^−1^)	Main product	Problem	Ref.
Organic additive	Na_2_C_6_O_6_	3.6	250/310	C_6_O_6_	Copious side effect	70 and 74
	Na_2_C_6_H_2_O_6_	3.98	250/265	Unknown	Limited sodium compensation and copious side effect	70
	CH_3_COONa	4.1	326/300	C_2_H_6_, CO_2_	High decomposition potential	71
	EDTA-4Na	3.79	282/420	CO, C_3_N, H_2_O, H_2_, O_2_	Produce flammable gas and H_2_O	76
	DTPA-5Na	3.61	266/363	CO, C_3_N, H_2_O, H_2_, O_2_	Produce flammable gas and H_2_O	72
	Na_2_C_4_O_4_	3.6	339/256	CO_2_, CO, C	Low electronic conductivity	73
	Na_2_C_2_O_4_	3.97	400/394	CO_2_	Low electronic conductivity	78
	Na_2_C_3_O_5_	4	331/310	C, CO_2_	Low electronic conductivity	79

### 2.6. Sodium-rich cathode materials

Although the sacrificial additive process is simple and convenient, it will inevitably generate some by-products (“dead mass”) and gases. They are prone to bring about safety problems and affect the subsequent cycles of the battery, limiting the practical application. Therefore, it has been proposed by some researchers that the direct use of some sodium-rich cathode materials can better realize sodium compensation, avoid adding other components in the cathode and decrease the negative effect of by-products.

**Fig. 8:**
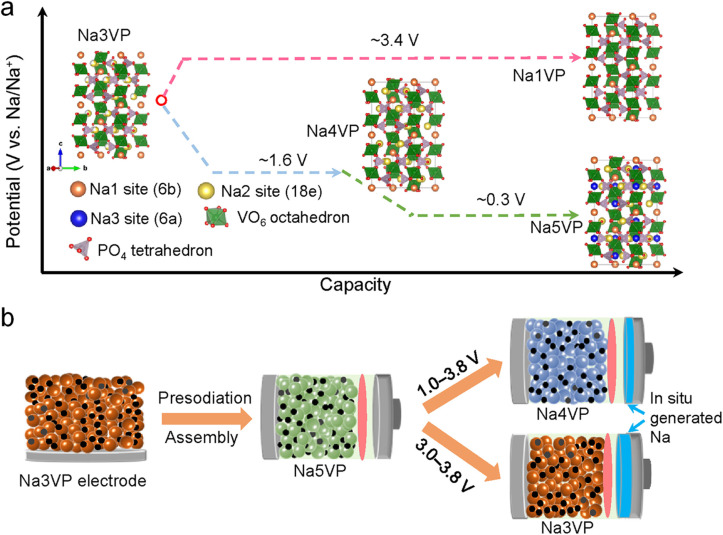
(a) Schematic illustration for phase transformations of Na_*x*_V_2_(PO_4_)_3_ and corresponding electrochemical potentials. (b) Illustration of sodium compensation on Na_*x*_V_2_(PO_4_)_3_, battery assembly, and battery operation within two different voltage windows. Reproduced with permission.^81^ Copyright 2022, Wiley-VCH.

Sodium Superionic Conductor (NASICON)-type phosphates have emerged as a promising cathode material candidate by virtue of the sodium-rich composition (2–5 Na species per chemical formula unit), fast ionic conductivity, and robust structure.^14,80^ Na_*x*_V_2_(PO_4_)_3_ stands out due to the rich redox couples of V (V^2+^/V^3+^, V^3+^/V^4+^, and V^4+^/V^5+^). As presented in Fig. 8a, the most representative V-based NASICON (*i.e.,* Na_3_V_2_(PO_4_)_3_, denoted as Na_3_VP) can be transformed to Na_4_VP and finally to Na_5_VP at 1.6 V and 0.3 V *versus* Na^+^/Na during the sodiation process.^82^ Each extra sodium ion in the formula can theoretically provide a capacity and 58.8 mA h g^−1^. Upon charging, Na_3_VP experiences a phase transition to Na_1_VP at 3.4 V *versus* Na^+^/Na and is the main phase during battery operation. Therefore, Na-rich phases including Na_4_VP and Na_5_VP are good choices as sodium compensation species for the sodium loss by HC anodes; after desodiation, the remaining Na_3_VP phase can reversibly function as a cathode (Na_3_VP ↔ Na_1_VP) in the subsequent cycles.

Jian *et al.*^83^ and Mirza *et al.*^84^ adopted an electrochemical SCT against a sodium metal electrode as mentioned in the above section to discharge Na_3_V_2_(PO_4_)_3_ into a sodium-rich Na_4_V_2_(PO_4_)_3_ cathode. The intermediate Na_4_VP phase serves as an extra sodium source to compensate for HC and as a cathode. The energy density of the HC‖Na_4_V_2_(PO_4_)_3_ full cell is 265 W h kg^−1^, which is 76% higher than that of the Na_3_VP counterpart. However, as discussed above, this electrochemical SCT is faced with complex disassembling, rinsing, and battery assembly processes, which is quite time-consuming and not appropriate for mass production.

Thus, Wu *et al.*^81^ employed a direct-contact SCT to achieve Na_4_VP and Na_5_VP Na-rich phases as shown in Fig. 8b. They assembled a full cell based on a sodium-free anode (Al/C collector) and Na_5_VP cathode, achieving a high energy density of 400 W h kg^−1^ and a high-capacity retention of 93% after 130 cycles. Although this method simplifies the technological process, it is still hard to be applied in practical application since the sodium compensation extent is difficult to control and sodium metal may give rise to safety hazards.

Alternatively, the chemical method using a Na complex holds great promise in this regard due to its straightforward operation, high efficiency, high safety, and easy scalability. Liu *et al.*^85^ subjected Na_3_VP to Na–Bp@DME for 30 s to realize a Na-rich Na_4_VP phase hinged on the large potential difference between Bp^−^/Bp (0.12 V *versus* Na^+^/Na) and sodiation of Na_3_VP (1.65 V *versus* Na^+^/Na). This approach endowed an anode-free full cell with much improved capacity and prolonged cycling performance. Nonetheless, the Na–Bp@DME reagent may not be an ideal reagent for generating Na_4_VP owing to the potential mismatch to incur an over-extent of sodium compensation to form Na_5_VP (0.3 V *versus* 0.12 V for the Bp^−^/Bp redox). To this end, Xu *et al.*^86^ further proposed phenazine-sodium (PNZ-Na) to achieve the sodiation of the Na_3_VP precursor to build sodium-rich Na_4_VP based on a redox-potential matching principle. PNZ-Na stands out because of its suitable reducing potential of 1.56 V *versus* Na^+^/Na, which is significantly higher than the sodium-rich potential of Na_5_VP (0.28 V *versus* Na^+^/Na) but is slightly lower than that of Na_4_VP (1.67 V *versus* Na^+^/Na). The spontaneous sodium reaction is able to rapidly insert one Na^+^ ion into the Na_3_VP framework, obtaining a phase-pure Na_4_VP within only 90 s. The obtained HC‖Na_4_VP full cell exhibited a high energy density of 251 W h kg^−1^, which was 58% higher than the 159 W h kg^−1^ of the pristine one.

The above evidence shows that sodium-rich cathode materials are promising for sodium compensation. But this type of SCT is quite limited since there are some V-based NASICON materials that have been proposed, and the sodium compensation is significantly based on the materials themselves, which is hard to be applied in other systems with different cathodes and anodes. More sodium-rich materials deserve to be explored and synthesized.

## 3 Summary and outlook

**Fig. 9:**
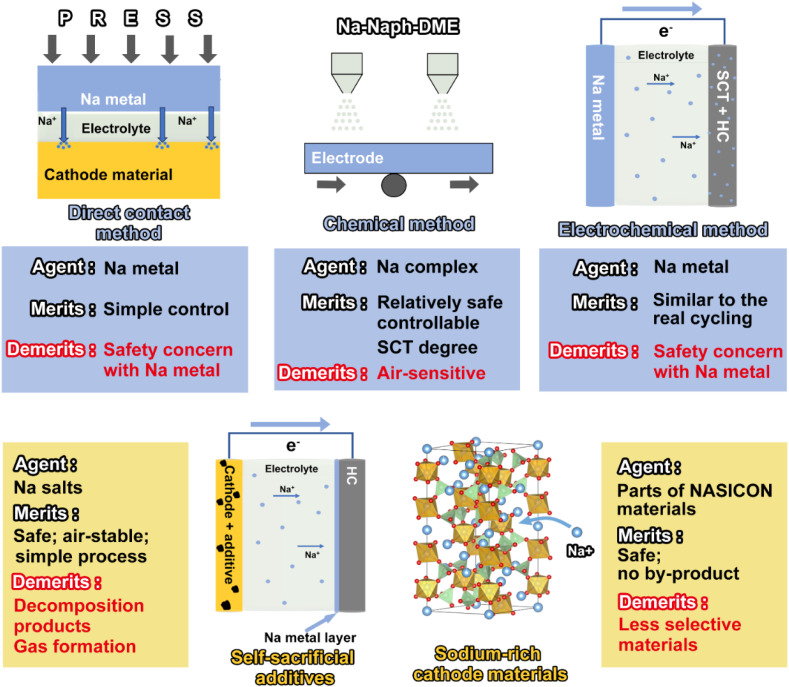
Schematic illustration of the merits, demerits, and operation of different sodium compensation technologies.

**Fig. 10:**
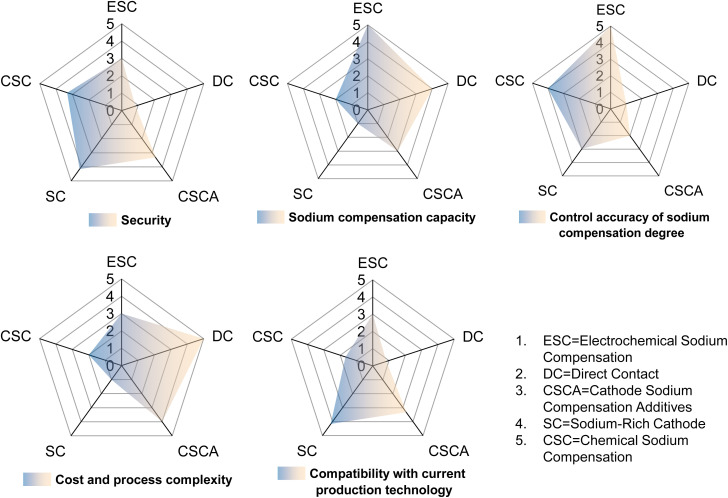
Key parameter radar chart of different SCTs.

Sodium-ion batteries, one of the most promising alternative technologies to lithium-ion batteries, have been constrained by a low initial coulombic efficiency and a low energy density. Sodium compensation technology has received widespread attention for its ability to effectively improve the ICE by releasing active sodium ions. This review systematically summarizes the up-to-date progress of sodium compensation technologies in recent years and provides an overview of the current most advanced sodium compensation strategies, aiming to provide researchers with a primary understanding of the significance and methods of sodium compensation. As shown in Table 4 and Fig. 9 and 10, different strategies have merits and demerits, and based on different material systems, the best means of sodium compensation remains to be selected for commercial production. Nonetheless, some challenges still require further research and optimization. Due to the high reactivity of sodium metal, which is prone to have chemical reactions with water and oxygen, sodium compensation strategies utilizing Na metal as a source of Na (electrochemical sodium compensation method, physical mixing method, and direct contact method) need to be carried out under strict anhydrous and oxygen-free conditions. Such a strict production environment leads to increased process costs. Furthermore, higher reactivity of Na metal means faster reaction times, which leads to difficulty in controlling the degree of sodium compensation and the formation of sodium dendrites, *etc*. And the larger ionic radius and different electrochemical behavior of sodium may affect the efficiency and longevity, while the control of the extent of sodium compensation as well as the process of disassembly and reassembly of the batteries restricts the practical application of the above technologies. Sodium-rich materials are efficient for sodium compensation and there is no additional component and any byproduct remaining in the battery. Nevertheless, this type of strategy is quite limited since there are nearly V-based NASICON materials reported and the sodium compensation is highly dependent on the materials themselves, which is hard to be applied in other systems with different cathodes and anodes. More sodium-rich materials deserve to be explored and synthesized.

**Table 4:** Merits and demerits of different sodium compensation technologies

Method	Agent	Merits	Demerits
Direct contact method	Metallic Na	Facile control; high sodium compensation capacity; low enough redox potential	Safety hazards with Na metal; sodium compensation extent difficulty
Electrochemical method	Metallic Na	Controllable SCT degree; comparable to the actual battery operation	Safety hazards with Na metal; complex process
Physical sodium compensation	Metallic Na	Facile control; high sodium compensation capacity; low enough redox potential	Safety hazards with Na metal
Chemical method	Na complex	Relatively safe; controllable SCT degree; simple process	Air-sensitive; insufficient redox potential
Self-sacrificial additives	Na salts	Safe; good stability; simple process	Gas formation; oxidative residues
Sodium-rich cathode materials	Parts of V-based NASICONs	Safe; no by-product	Less selective materials; low SCT capacity

In contrast, chemical sodium compensation and self-sacrificial additives are the most promising sodium compensation technologies by virtue of their simple and safe processes and controllable degree of sodium compensation. Overall, the strategies remain at the academic level for further exploration from the laboratory to industrialization.

The chemical sodium compensation method should further consider: (i) the electrode of industrial production is characterized by high loading and low porosity, which is not suitable for the wetting of the material by the sodium compensation solution, leading to inconsistency in the extent of sodium compensation at different positions of the electrode. Therefore, it is necessary to further systematically investigate how to realize the accurate control of sodium compensation. (ii) The SCT-treated electrode material has a low open-circuit potential and a strong “electronegativity”, resulting in inferior air stability. How to achieve a long-time storage of electrodes in air needs to be solved urgently. (iii) The impact of residual sodium compensation reagent on the subsequent battery performance remains to be clearly analyzed. (iv) Recovering and reusing the sodium compensation reagent and organic reagent can effectively reduce the production cost and protect the environment. (v) The safety issue should be paid more attention. The usually employed ether solvents possess a quite low flash point and boiling point. Ideal SCT solvents need to be further explored and designed at a molecular level with a high enough boiling point and flash point as well as an excellent compatibility with the Na complex. (vi) The currently used sodium reagents are hinged on polycyclic aromatic hydrocarbons with a low reducing potential (∼0.1 V *versus* Na^+^/Na), easily incurring sodium accumulation and causing the electrode's structure to deteriorate. How to modulate the reducing potential of the Na-complex to a suitable range remains to be further studied by *in situ* experimental characterization and theoretical simulations.

The large-scale utilization of sodium cathode compensation additives remains to solve the following problems: (i) the additive should have good air stability to avoid rapid reaction with water and oxygen. (ii) To reduce the gas generated by the decomposition of sodium compensation additives and the impact of changes in the electrode interface caused by the gas in the follow-up cycle of the battery, to prevent the decomposition products from continuing to participate in the subsequent reaction. (iii) To improve the irreversible capacity of the sodium compensation additive, to reduce its voltage polarization, and to reduce the amounts of additives in the electrodes. (iv) The operating mechanism of sodium compensation additives lacks in-depth study. There are many side reactions, and the effects on the composition and structure of SEI and CEI layers are not clear. (v) The oxidative potential of these additives should be elaborately lowered from the perspective of catalysis within the safe operating voltage range of electrolytes to prevent the over-decomposition of the electrolyte. (vi) The residual will become the “dead mass” to lower the overall energy density. Some *in situ* sodium compensation methods can be taken into account. For instance, the residual alkali (*e.g.*, NaOH, Na_2_O, Na_2_CO_3_, *etc.*) around the cathode surface can be neutralized by some organic acids (*e.g.*, acetic acid) to form a sodium organic salt as an efficient sodium compensation additive for subsequent battery operation.

In the section that follows, we also offer ideas and prospects for the advancement of SCTs in the future, addressing issues with high-throughput screening, characterization, manufacturing, equipment, and recycling.

(1) High-throughput screening. In recent years, high-throughput screening has become more crucial in the investigation of novel materials due to the advancement of high-throughput computing based on DFT. By determining the LUMO–HOMO energy level of solvents, additives, and electrolyte systems, new solvents for chemical sodium compensation and self-sacrificial sodium additives utilized in cathodes can be produced for sodium compensation. It will select a large number of chemical reagents that satisfy the fundamental parameter requirements. Some of these reagents will then be sent for higher-order parameter refinement and characterization, and machine learning will be used to analyze the correlation between the calculated data and the characterization results, thereby offering a trustworthy framework for the development of new sodium compensation reagents.

(2) New characterization. As of right now, no characterization technology is able to more accurately represent the sodium compensation process. The advancement of sodium compensation research is substantially slowed down by the fact that studies on the subject exclusively rely on electron microscope images and electrochemical performance data. Therefore, based on current electrochemical theory, it is imperative to develop new characterization approaches that can directly or indirectly indicate the sodium compensation effect.

(3) Manufacturing and equipment. Sodium compensation process control equipment design is especially crucial for large-scale production of SCTs. It can keep an eye on the condition of the electrode materials during the sodium compensation process and provide real-time feedback to the terminal equipment to make adjustments. This allows for automated and precise management of the sodium compensation degree while also improving safety.

(4) Recycling. The large-scale implementation of sodium compensation materials/reagent recycling in SIBs holds significant strategic importance for the future. Recycling sodium compensation materials and/or chemicals promotes a safer and more robust raw material supply chain in addition to easing the restrictions brought on by a shortage of raw materials and improving environmental sustainability. In the meantime, they have a higher added value for recycling and are fundamentally distinct from other conventional electrode materials.

In summary, we believe that sodium compensation is a critical approach that needs to be included in the design of the materials and electrolytes. Doing so will undoubtedly help to improve SIBs in the future.

## Data availability

The type of this manuscript is a review and related data are cited from other sources. Therefore, there are no raw experimental or computational data associated with this article.

## Author contributions

W. Zhang, B. Zhu, and Z. Zhang designed the structure of the review. W. Zhang, B. Zhu, Z. Jiang, J. Chen, Z. Li, J. Zheng, and N. Wen contributed to the illustration. W. Zhang and B. Zhu collected the papers related to this review topic and co-wrote the paper. W. Zhang and Z. Zhang supervised this project. All authors discussed and revised the manuscript.

## Conflicts of interest

The authors declare no competing interests.
